# Enhanced Calibration Method for Robotic Flexible 3D Scanning System †

**DOI:** 10.3390/s25154661

**Published:** 2025-07-27

**Authors:** Zhilong Zhou, Jinyong Shangguan, Xuemei Sun, Yunlong Liu, Xu Zhang, Dengbo Zhang, Haoran Liu

**Affiliations:** College of Mechanical and Vehicle Engineering, Linyi University, Linyi 276012, China; shangguanjinyong@lyu.edu.cn (J.S.); 17861279067@163.com (Y.L.); 17753854071@163.com (X.Z.); zhangdengbo@lyu.edu.cn (D.Z.); 18763329293@163.com (H.L.)

**Keywords:** robotic 3D scanning system, hand-eye calibration, kinematic parameter calibration, geometric constraints, error correction

## Abstract

Large-sized components with numerous small key local features are essential in advanced manufacturing. Achieving high-precision quality control necessitates accurate and highly efficient three-dimensional (3D) measurement techniques. A flexible measurement system integrating a fringe-projection-based 3D scanner with an industrial robot is developed to enable the rapid measurement of large object surfaces. To enhance overall measurement accuracy, we propose an enhanced calibration method utilizing a multidimensional ball-based calibrator to simultaneously calibrate for hand-eye transformation and robot kinematic parameters. Firstly, a preliminary hand-eye calibration method is introduced to compensate for measurement errors at observation points, leveraging geometric-constraint-based optimization and a virtual single point derived via the barycentric calculation method. Subsequently, a distance-constrained calibration method is proposed to jointly estimate the hand-eye transformation and robot kinematic parameters, wherein a distance error model is constructed to link parameter errors with the measured deviations of a virtual single point. Finally, calibration and validation experiments were carried out, and the results indicate that the maximum and average measurement errors were reduced from 1.053 mm and 0.814 mm to 0.421 mm and 0.373 mm, respectively, thereby confirming the effectiveness of the proposed method.

## 1. Introduction

Complicated components with numerous small key local features (KLFs) are commonly used in modern advanced manufacturing industries such as aerospace, marine engineering, and automotive sectors. These features, including edges, holes, grooves, and curved surfaces, often exhibit complex geometries and tight tolerances, which pose significant challenges for dimensional inspection and quality assurance. Automated and accurate 3D shape measurement of these features is critical for ensuring quality control, enhancing product reliability, and minimizing manufacturing costs [1,2,3]. As product designs become increasingly complex and lightweight, the demand for high-precision and intelligent inspection technologies continues to grow, driving the need for more effective measurement systems capable of handling diverse geometries under varying operational conditions.

Three-dimensional (3D) shape measurement techniques are generally classified into contact and non-contact approaches. Among contact-based methods, coordinate measuring machines (CMMs) equipped with tactile probes [4] offer exceptional precision. Nevertheless, they are constrained by low efficiency in acquiring high-density 3D point cloud data. With the continued advancement of optical non-contact measurement technologies, line-structured light sensors based on active vision have been widely applied in industrial visual inspection and 3D shape measurement due to their non-contact nature, high precision, and efficiency [5]. However, these sensors have limited fields of view and lack intrinsic motion capabilities, making them unsuitable for measuring the full geometry of large-scale objects. To address this, they are typically mounted on high-precision motion platforms to enable large-area, multi-angle, and full-coverage measurements [6,7]. In recent years, structured light sensors have been widely used in industrial applications and integrated with CMMs for 3D shape measurements. However, the combined method, which integrates CMMs with a structured light sensor [8], is not suitable for online measurements due to its restrictive mechanical structure and limited measurement efficiency. In contrast to CMMs, industrial robots are well-suited for executing complex spatial positioning and orientation tasks with efficiency [9,10]. Equipped with a high-performance controller, these robots can also transport a vision sensor mounted on the end-effector to specified target locations. During the online measurement process, the spatial pose of the vision sensor varies as the robot’s end-effector moves to different positions. Nevertheless, the relative transformation between the sensor’s coordinate system and the end-effector remains fixed-an essential concept known as hand-eye calibration. Consequently, the accuracy of this calibration plays a pivotal role in determining the overall measurement accuracy of the system.

For hand-eye calibration, techniques are generally classified into three categories based on the nature of the calibration object: 2D target-based methods, single-point or standard-sphere-based methods, and 3D-object-based methods. One of the most widely recognized hand-eye calibration methods based on a two-dimensional calibration target was introduced by Shiu [11]. The spatial relationship between the sensor and the robot’s wrist frame was identified by analyzing the sensor’s motion in response to controlled movements of the robotic arm, typically modeled using the hand-eye calibration equation AX = XB, where A and B represent the robot and sensor transformations, respectively, and X denotes the unknown sensor pose. Under general conditions, this equation has a closed-form solution, provided that at least two distinct robot motions are used to resolve the inherent ambiguities in rotation and translation. The uniqueness of the solution depends on the rotational characteristics of the robot’s movements. Since then, a wide range of solution methods have been developed for these calibration equations, which are generally categorized into linear and nonlinear algorithms [12,13]. Representative approaches include the distributed closed-loop method, the global closed-loop method, and various iterative techniques. However, these methods tend to be highly sensitive to measurement noise. In contrast to traditional hand-eye calibration methods, which often involve complex setups and labor-intensive manual procedures, Sung et al. [14] proposed a vision-based self-calibration method tailored for industrial robotic applications. The approach utilizes a static 2D calibration plane and a camera mounted on the end-effector of a moving robot. By designing controlled translational and rotational movements of the robot arm, the spatial relationship between the robot and the camera can be accurately estimated without external intervention. This self-calibration strategy significantly reduces human involvement and enhances the practicality of deploying robotic vision systems in automated environments. To streamline the calibration process for line-structured light sensors in robotic eye-in-hand systems, Wang et al. [15] introduced an integrated method that simultaneously determines the camera intrinsics, hand-eye transformation, and structured light plane parameters. Unlike traditional approaches that treat each parameter group separately, often resulting in complex and time-consuming workflows, this method performs all calibrations using a unified image set acquired from multiple robot and target poses. By reusing the extrinsic results from camera calibration, the structured light plane and hand-eye parameters can be efficiently derived without additional data collection. The procedure requires only a low-cost planar checkerboard as the calibration target, making it both practical and accessible. Experimental results demonstrated that the method achieves sub-millimeter accuracy with a total calibration time under 10 min, validating its effectiveness for industrial 3D measurement applications. Pavlovčič et al. [16] proposed an efficient calibration method that simultaneously estimates both the intrinsic parameters of a laser profilometer and the hand-eye transformation with respect to an industrial robot. Unlike conventional approaches that often rely on multiple calibration targets or sequential procedures, their method requires only a single reference geometry scanned from multiple viewpoints. By capturing the reference object from 15 different poses and numerically optimizing the transformation parameters to minimize geometric deviations, the approach achieves high accuracy with minimal setup. Experimental validation demonstrated sub-millimeter calibration precision, achieving deviations below 0.105 mm when using a robotic arm and 0.046 mm with a CNC system. The fully automated workflow, which completes in under 10 min, makes this approach highly suitable for regular on-site recalibration in industrial environments. In contrast, Xu et al. [17] proposed a single-point-based calibration method that utilizes a single point, such as a standard sphere, to compute the transformation, offering a relatively simple and practical implementation. Furthermore, hand-eye calibration methods utilizing a standard sphere of known radius have been extensively adopted to estimate the hand-eye transformation parameters [18]. These methods provide an intuitive and user-friendly solution for determining both rotation and translation matrices. Among the 3D-object-based calibration methods, Liu et al. [19] designed a calibration target consisting of a small square with a circular hole. Feature points were extracted from line fringe images and used as reference points for the calibration process. However, the limited number of calibration points and the low precision of the extracted image features resulted in suboptimal calibration accuracy. In addition to hand-eye calibration methods based on calibration objects, several approaches that use environmental images, rather than calibration objects, have been successfully applied to determine hand-eye transformation parameters, as reported by Sang [20] and Nicolas and Qi [21]. To enhance the flexibility and autonomy of 3D measurement systems, Song et al. [22] proposed a hand-eye calibration method that avoids reliance on high-precision or specially designed calibration targets. Instead, their approach leverages irregular and unstructured objects, capturing 3D point cloud data from multiple viewpoints to estimate the spatial relationship between the robot and the sensor. By integrating a fast point feature histogram (FPFH)-based sampling consistency check with an improved iterative closest point registration algorithm that incorporates probabilistic modeling and covariance refinement, the method establishes the hand-eye transformation through robust point cloud alignment. Experimental results confirmed that the technique can perform accurate calibration using arbitrary objects in real-world robotic applications. However, the methods mentioned above do not account for the robot positioning errors introduced by kinematic parameter errors during the hand-eye calibration process.

To overcome this limitation, many researchers have proposed various hand-eye calibration methods that account for the correction of robot kinematic parameter errors. Yin et al. [23] introduced an enhanced hand-eye calibration algorithm. Initially, hand-eye calibration is conducted using a standard sphere, without accounting for robot positioning errors. Then, both the robot’s kinematic parameters and the hand-eye relationship parameters are iteratively refined based on differential motion theory. Finally, a unified identification of hand-eye and kinematic parameter errors is accomplished using the singular value decomposition (SVD) method. However, the spherical constraint-based error model lacks absolute dimensional information, and cumulative sensor measurement errors further limit the accuracy of parameter estimation. Li et al. [24] introduced a method that incorporates fixed-point information as optimization constraints to concurrently estimate both hand-eye transformation and robot kinematic parameters. However, the presence of measurement errors from visual sensors substantially compromises the robustness and generalizability of the resulting parameter estimations. To further optimize measurement system parameters, Mu et al. [25] introduced a unified calibration method that simultaneously estimates hand-eye and robot kinematic parameters based on distance constraints. However, this method does not adequately address sensor measurement error correction during the solution of the hand-eye matrix, resulting in accumulated sensor inaccuracies that significantly degrade the precision of the derived relationship matrix.

To address the aforementioned limitations, we propose an accurate calibration method for a robotic flexible 3D scanning system based on a multidimensional ball-based calibrator (MBC) [26]. This method constructs a distance-based calibration model that concurrently considers measurement errors, hand-eye parameter errors, and robotic kinematic errors. Specifically, by incorporating geometric-constraint-based optimization for compensating coordinate errors of the measurement points, a preliminary hand-eye calibration method is introduced based on a single virtual point determined via the barycenter technique. Subsequently, a distance-constraint-based calibration method is developed to further optimize both hand-eye and kinematic parameters, effectively associating system parameter errors with deviations in the measured coordinates of the single virtual point.

The remainder of this paper is organized as follows. Section 2 introduces the model of the measurement system. Section 3 outlines the proposed calibration methodology. Section 4 presents the experimental setup and accuracy validation. Finally, Section 5 concludes this study with a brief summary of the findings.

## 2. Measurement Method and Principle

### 2.1. Measurement Method

**Figure 1:**
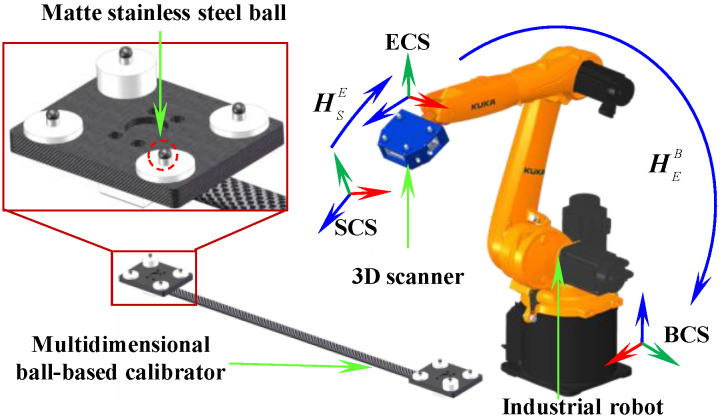
Schematic of robotic flexible 3D scanning system.

The proposed robotic 3D scanning system integrates an industrial robot with a fringe-projection-based 3D scanner, which is mounted on the robot’s end-effector using a custom fixture. For accurate system calibration and validation, a specially designed multidimensional ball-based calibrator (MBC) is utilized. The MBC features eight non-collinear spheres, whose spatial relationships are pre-calibrated using a CMM. As illustrated in Figure 1, the system comprises three coordinate frames: the robot base coordinate system $O_{b}-X_{b}Y_{b}Z_{b}$ (BCS), the robot end-effector coordinate system $O_{e}-X_{e}Y_{e}Z_{e}$ (ECS), and the 3D scanner coordinate system $O_{s}-X_{s}Y_{s}Z_{s}$ (SCS).

During the measurement process, the robot adjusts its pose to guide the scanner in acquiring the target features. The acquired data are subsequently transformed from SCS to BCS. Based on the coordinate transformation principle, the measured coordinates of the visual points PcS in the SCS can be expressed PcB in the BCS is as follows:

(1) PcB1=REBTEB01RSETSE01PcS1

where $R_{E}^{B}$ and $T_{E}^{B}$ represent the rotation matrix and translation vector, respectively, from ECS to BCS, while $R_{S}^{E}$ and $T_{S}^{E}$ represent the rotation matrix and translation vector, respectively, in the hand-eye relation.

To ensure accurate measurements throughout the system, three calibration procedures must be completed prior to data acquisition: (1) intrinsic calibration of the 3D sensor, (2) hand-eye calibration, and (3) calibration of the robot’s kinematic parameters.

In this study, the intrinsic parameters of the 3D scanner were pre-calibrated and verified by the manufacturer. Therefore, they are assumed to remain constant during operation. Consequently, only the hand-eye calibration and the robot kinematic calibration are required, as further described in Section 3.

### 2.2. Robot Kinematic Error Model

Due to structural and manufacturing imperfections, deviations arise between a robot’s actual and nominal kinematic parameters, leading to discrepancies in its actual and theoretical poses—commonly referred to as positioning errors. A kinematic error model quantitatively characterizes the relationship between these parameter deviations and the resulting positioning errors. In the study, the error model is formulated based on the Modified Denavit-Hartenberg (MD-H) convention [27] and rigid-body differential motion theory [28] and is expressed as follows:

(2) $$H_{E}^{B}+dH_{E}^{B}=\prod_{i=1}^{N}\left(H_{i}^{i-1}+dH_{i}^{i-1}\right)$$

where $dH_{E}^{B}$ is the deviation in the homogenous transformation matrix between ECS and BCS, and $H_{i}^{i-1}$ is the homogenous transformation matrix between the adjacent joints.

By expanding Equation (2) and neglecting the second-order term and combining it with the differential kinematics model $dH_{E}^{B}=H_{E}^{B}\delta H_{E}^{B}$, we obtain

(3) $$\begin{array}{l} H_{E}^{B}+dH_{E}^{B}=H_{E}^{B}+H_{E}^{B}\delta H_{E}^{B}\\ \;\;\;\;=H_{E}^{B}+\sum_{i=1}^{n}\left(\frac{\partial H_{E}^{B}}{\partial \theta_{i}}\Delta \theta_{i}+\frac{\partial H_{E}^{B}}{\partial d_{i}}\Delta d_{i}+\frac{\partial H_{E}^{B}}{\partial a_{i}}\Delta a_{i}+\frac{\partial H_{E}^{B}}{\partial \alpha_{i}}\Delta \alpha_{i}+\frac{\partial H_{E}^{B}}{\partial \beta_{i}}\Delta \beta_{i}\right) \end{array}$$

where $\Delta \theta_{i}$, $\Delta d_{i}$, $\Delta a_{i}$, $\Delta \alpha_{i}$, and $\Delta \beta_{i}$ are small link parameter errors.

Consequently, the relationship between the robot positioning error and kinematic parameter errors can be expressed as

(4) $$\Delta E=\left[\begin{array}{l} \Delta D\\ \Delta \Theta \end{array}\right]=\left[\begin{array}{l} M_{1}\\ M_{2} \end{array}\right]\Delta \theta +\left[\begin{array}{l} M_{2}\\ 0 \end{array}\right]\Delta d+\left[\begin{array}{l} M_{3}\\ 0 \end{array}\right]\Delta a+\left[\begin{array}{l} M_{4}\\ M_{3} \end{array}\right]\Delta \alpha +\left[\begin{array}{l} M_{5}\\ M_{6} \end{array}\right]\Delta \beta =J\Delta X\;\;$$

where $\Delta E={\left[\Delta e_{x}\;\Delta e_{y}\;\Delta e_{z}\;\;\delta e_{x}\;\delta e_{y}\;\delta e_{z}\right]}^{T}$ and $\Delta X={\left[\Delta \theta \;\Delta d\;\Delta a\;\right.\left.\Delta \alpha \;\Delta \beta \right]}^{T}$ and ΔD and ΔΘ represent, respectively, the differential translation vector and differential rotation vector; Δθ, Δd, Δa, Δα, and Δβ represent the kinematic parameter errors vector; $M_{1}∼M_{6}$ are the 3×n error coefficient matrices; and ***J*** is the Jacobian matrix.

## 3. Measurement System Calibration

**Figure 2:**
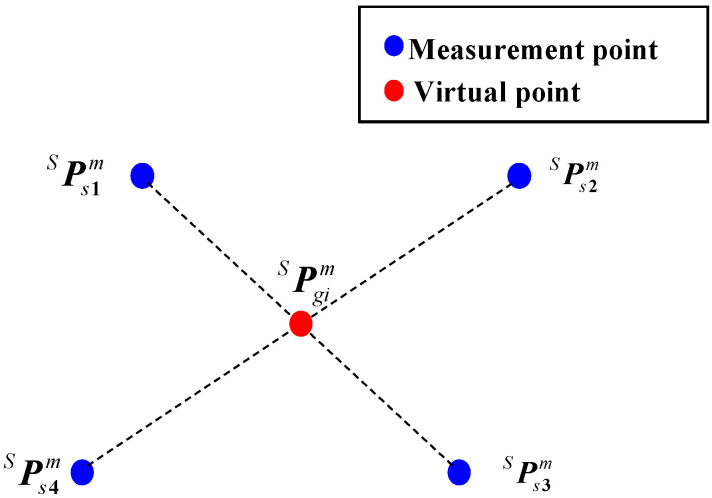
Diagram of the single virtual point.

To improve the accuracy of hand-eye calibration, this paper proposed an online accurate calibration method using a multidimensional ball-based calibrator (MBC) to simultaneously identify both hand-eye transformation and robot kinematic parameters. A schematic of the calibration procedure is presented in Figure 2. First, Section 3.1 introduces an initial hand-eye calibration method that compensates for measurement point errors using an angular-constraint-based optimization method. This method is based on a virtual point calculated via the barycenter algorithm. Subsequently, Section 3.2 presents a distance-constraint-based calibration strategy to further refine the estimation of both hand-eye transformation and kinematic parameters.

### 3.1. Initial Calibration of the Hand-Eye Parameters

#### 3.1.1. Preliminary Hand-Eye Calibration Method Based on a Virtual Single Point

During the initial stage of hand-eye calibration, the MBC is stably positioned within a suitable workspace. A single virtual point, serving as the calibration target, is calculated based on the coordinates of four measurement points optimized using an angular-constraint-based optimization method. Specifically, the initial hand-eye transformation is then determined by capturing this virtual point from multiple robots’ poses using the 3D scanner. Furthermore, the position of the single virtual point relative to the BCS remains constant. According to the principle of barycentric coordinates, an initial (non-optimized) virtual point PgimS=xgimS,ygimS,zgimST, as illustrated in Figure 2, is computed based on the initial coordinates of four measurement points PsimS=xsimS,ysimS,zsimST and is formulated as follows:

(5) PgimS=14∑i=14xsimS,14∑i=14ysimS,14∑i=14zsimST

When the 3D scanner moves to the *i*-th and *j*-th poses, the following equations, considering the measurement deviation, can be obtained according to Equation (1):

(6) PgiB=RE0iBRSEPgimS+ΔPgimS+RE0iBTSETE0iBPgjB=RE0jBRSEPgjmS+ΔPgjmS+RE0jBTSETE0jB

where ΔPgimS, computed by the optimized method introduced in Section 3.1.2, is the deviation between the nominal and actual coordinates of the single virtual point in the SCS.

By translating the 3D scanner multiple times to measure the points, a matrix equation in the form of $R_{S}^{E}A=b$ is given by

(7) RSEPg1mS+ΔPg1mS−Pg2mS+ΔPg2mS⋮Pg1mS+ΔPg1mS−PgnmS+ΔPgnmST=RE0iBTTE02B−TE02B⋮TE0nB−TE02BT

The unknown matrix $R_{S}^{E}$ is determined using the singular value decomposition (SVD) algorithm. Subsequently, the unknown translation vector $T_{S}^{E}$ is calculated via the least squares method. Since measurement errors at the target points substantially affect the overall calibration accuracy, it is essential to optimize the coordinates of the measurement points acquired by the 3D scanner.

#### 3.1.2. Measurement Error Identification Method

**Figure 3:**
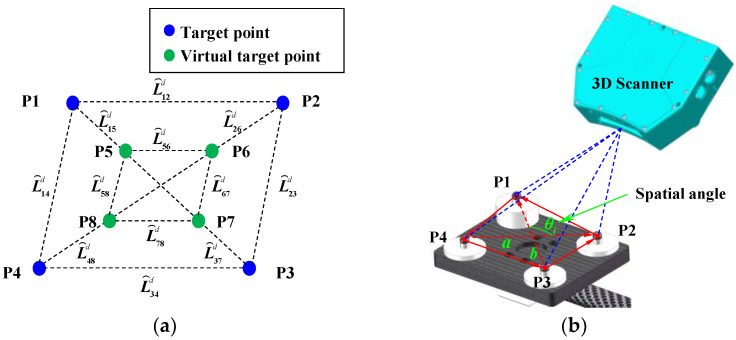
Diagram of prior geometric knowledge: (**a**) diagram of constrained distances constructed by target points and (**b**) diagram of a spatial angle formed by two nonzero vectors.

To identify and minimize measurement errors at the target points, an adjustment optimization method is introduced in which prior geometric knowledge is used to construct distance and angular constraints among the target points. The constrained distances constructed by different target points and the spatial angle formed by two nonzero vectors are illustrated in Figure 3a and Figure 3b, respectively.

First, a coordinate measurement optimization model based on distance constraints is constructed to perform an initial correction of measurement errors at the target points. Let $P^{m}=\left[x_{1}^{m},y_{1}^{m},\cdots ,y_{n}^{m},z_{n}^{m}\right]$ and ${\overset{⌢}{P}}^{r}=\left[{\overset{⌢}{x}}_{1}^{r},{\overset{⌢}{y}}_{1}^{r},\cdots ,{\overset{⌢}{y}}_{n}^{r},{\overset{⌢}{z}}_{n}^{r}\right]$ represent the three-dimensional coordinate measurement values and theoretical values of the target points on the MBC obtained by the 3D scanner, respectively. Then, a virtual target point is determined by computing the centroid of three target points, and together with the coordinates of all points, distance constraints are constructed. The theoretical distance value ${\overset{⌢}{L}}_{ij}^{d}$ between any two target points and their theoretical coordinate values has the following relationship:

(8) $$\left\{\begin{array}{c} {\left({\overset{⌢}{x}}_{1}^{r}-{\overset{⌢}{x}}_{2}^{r}\right)}^{2}+{\left({\overset{⌢}{y}}_{1}^{r}-{\overset{⌢}{y}}_{2}^{r}\right)}^{2}+{\left({\overset{⌢}{z}}_{1}^{r}-{\overset{⌢}{z}}_{2}^{r}\right)}^{2}-{\left({\overset{⌢}{L}}_{12}^{D}\right)}^{2}=0\\ \vdots \\ {\left({\overset{⌢}{x}}_{i}^{r}-{\overset{⌢}{x}}_{j}^{r}\right)}^{2}+{\left({\overset{⌢}{y}}_{i}^{r}-{\overset{⌢}{y}}_{j}^{r}\right)}^{2}+{\left({\overset{⌢}{z}}_{i}^{r}-{\overset{⌢}{z}}_{j}^{r}\right)}^{2}-{\left({\overset{⌢}{L}}_{ij}^{D}\right)}^{2}=0\\ \vdots \\ {\left({\overset{⌢}{x}}_{n-1}^{r}-{\overset{⌢}{x}}_{n}^{r}\right)}^{2}+{\left({\overset{⌢}{y}}_{n-1}^{r}-{\overset{⌢}{y}}_{n}^{r}\right)}^{2}+{\left({\overset{⌢}{z}}_{n-1}^{r}-{\overset{⌢}{z}}_{n}^{r}\right)}^{2}-{\left({\overset{⌢}{L}}_{n\;\left(n-1\right)}^{D}\right)}^{2}=0 \end{array}\right.$$

Assuming that

(9) $$f_{d}=\sqrt{{\left({\overset{⌢}{x}}_{i}^{r}-{\overset{⌢}{x}}_{j}^{r}\right)}^{2}+{\left({\overset{⌢}{y}}_{i}^{r}-{\overset{⌢}{y}}_{j}^{r}\right)}^{2}+{\left({\overset{⌢}{z}}_{i}^{r}-{\overset{⌢}{z}}_{j}^{r}\right)}^{2}}\left(i,j=1,2,\cdots ,n;i\neq j\right)$$

Performing a Taylor expansion on the above equation and ignoring the terms of higher order than the second, we obtain the linearized equation of the distance constraint:

(10) $$f_{d}=L_{ij}^{m}+\frac{\partial f_{d}}{\partial {\overset{⌢}{x}}_{i}^{r}}\delta {\overset{⌢}{x}}_{i}^{m}+\frac{\partial f_{d}}{\partial {\overset{⌢}{y}}_{i}^{r}}\delta {\overset{⌢}{y}}_{i}^{m}+\frac{\partial f_{d}}{\partial {\overset{⌢}{z}}_{i}^{r}}\delta {\overset{⌢}{z}}_{i}^{m}+\frac{\partial f_{d}}{\partial {\overset{⌢}{x}}_{j}^{r}}\delta {\overset{⌢}{x}}_{j}^{m}+\frac{\partial f_{d}}{\partial {\overset{⌢}{y}}_{j}^{r}}\delta {\overset{⌢}{y}}_{j}^{m}+\frac{\partial f_{d}}{\partial {\overset{⌢}{z}}_{j}^{r}}\delta {\overset{⌢}{z}}_{j}^{m}$$

where $L_{ij}^{m}=\sqrt{{\left(x_{i}^{m}-x_{j}^{m}\right)}^{2}+{\left(y_{i}^{m}-y_{j}^{m}\right)}^{2}+{\left(z_{i}^{m}-z_{j}^{m}\right)}^{2}}$, which is the measurement value of the distance between two target points; $\delta {\overset{⌢}{X}}^{m}={\left[\delta {\overset{⌢}{x}}_{i}^{m},\delta {\overset{⌢}{y}}_{i}^{m},\delta {\overset{⌢}{z}}_{i}^{m},\delta {\overset{⌢}{x}}_{j}^{m},\delta {\overset{⌢}{y}}_{j}^{m},\delta {\overset{⌢}{z}}_{j}^{m}\right]}^{T}$ is the correction vector for measurement errors of the two target points on site; $\left(\frac{\partial f_{d}}{\partial {\overset{⌢}{x}}_{i}^{r}},\frac{\partial f_{d}}{\partial {\overset{⌢}{y}}_{i}^{r}},\frac{\partial f_{d}}{\partial {\overset{⌢}{z}}_{i}^{r}},\frac{\partial f_{d}}{\partial {\overset{⌢}{x}}_{j}^{r}},\frac{\partial f_{d}}{\partial {\overset{⌢}{y}}_{j}^{r}},\frac{\partial f_{d}}{\partial {\overset{⌢}{z}}_{j}^{r}}\right)$ is the partial derivative:

(11) $$\left\{\begin{array}{c} {\left(\frac{\partial f_{d}}{\partial {\overset{⌢}{x}}_{i}^{r}}\right)}_{0}=-\frac{x_{j}^{m}-x_{i}^{m}}{L_{ij}^{m}}=-\frac{\Delta x_{ij}^{0}}{L_{ij}^{m}}\\ {\left(\frac{\partial f_{d}}{\partial {\overset{⌢}{y}}_{i}^{r}}\right)}_{0}=-\frac{y_{j}^{m}-y_{i}^{m}}{L_{ij}^{m}}=-\frac{\Delta y_{ij}^{0}}{L_{ij}^{m}}\\ {\left(\frac{\partial f_{d}}{\partial {\overset{⌢}{z}}_{i}^{r}}\right)}_{0}=-\frac{z_{j}^{m}-z_{i}^{m}}{L_{ij}^{m}}=-\frac{\Delta z_{ij}^{0}}{L_{ij}^{m}} \end{array}\right.,\;\left\{\begin{array}{c} {\left(\frac{\partial f_{d}}{\partial {\overset{⌢}{x}}_{j}^{r}}\right)}_{0}=-\frac{x_{i}^{m}-x_{j}^{m}}{L_{ij}^{m}}=\frac{\Delta x_{ij}^{0}}{L_{ij}^{m}}\\ {\left(\frac{\partial f_{d}}{\partial {\overset{⌢}{y}}_{j}^{r}}\right)}_{0}=-\frac{y_{i}^{m}-y_{j}^{m}}{L_{ij}^{m}}=\frac{\Delta y_{ij}^{0}}{L_{ij}^{m}}\\ {\left(\frac{\partial f_{d}}{\partial {\overset{⌢}{z}}_{j}^{r}}\right)}_{0}=-\frac{z_{i}^{m}-z_{j}^{m}}{L_{ij}^{m}}=\frac{\Delta z_{ij}^{0}}{L_{ij}^{m}} \end{array}\right.$$

Further, we obtain

(12) $$\frac{\Delta x_{ij}^{0}}{L_{ij}^{m}}\delta {\overset{⌢}{x}}_{i}^{m}+\frac{\Delta y_{ij}^{0}}{L_{ij}^{m}}\delta {\overset{⌢}{y}}_{i}^{m}+\frac{\Delta z_{ij}^{0}}{L_{ij}^{m}}\delta {\overset{⌢}{z}}_{i}^{m}-\frac{\Delta x_{ij}^{0}}{L_{ij}^{m}}\delta {\overset{⌢}{x}}_{j}^{m}-\frac{\Delta y_{ij}^{0}}{L_{ij}^{m}}\delta {\overset{⌢}{y}}_{j}^{m}-\frac{\Delta z_{ij}^{0}}{L_{ij}^{m}}\delta {\overset{⌢}{z}}_{j}^{m}+l_{ij}^{D}=0$$

where $l_{ij}^{d}=-\left(L_{ij}^{m}-{\overset{⌢}{L}}_{ij}^{d}\right)$ is the closure error.

Thus, the matrix form of Equation (12) can be organized as follows:

(13) $$A^{d}\delta {\overset{⌢}{X}}^{m}-\delta L^{d}=0$$

where $A^{d}$ is the coefficient matrix; $\delta L^{d}={\left[l_{12}^{d},l_{13}^{d},\cdots ,l_{\left(n-1\right)n}^{d}\right]}^{T}$ is the closure error vector.

In Equation (13), there are 3*n* unknowns and $\frac{n\left(n-1\right)}{2}$ equations. According to the Lagrange multiplier method of conditional extremum, the objective function is obtained as

(14) $$\Phi^{d}={\left(\delta {\overset{⌢}{X}}^{m}\right)}^{T}P^{d}\delta {\overset{⌢}{X}}^{m}-2{\left(K^{d}\right)}^{T}\left(A^{d}\delta {\overset{⌢}{X}}^{m}-\delta L^{d}\right)$$

where $K^{d}={\left[k_{1}^{d},k_{2}^{d},\ldots ,k_{n}^{d}\right]}^{T}$ is the contact number vector.

By taking the first derivative of $\delta {\overset{⌢}{X}}^{m}$ and making it zero, therefore, in conclusion, we can obtain the optimized results of the observed values:

(15) $${\overset{⌢}{X}}^{d}=P^{m}+\delta {\overset{⌢}{X}}^{m}$$

Subsequently, a coordinate measurement optimization model based on angular constraints is established to further enhance the correction of measurement errors at the target points. Based on the property that the angle between two vectors in Euclidean space is independent of the coordinate system, the angle values formed by the target points on the MBC are considered as the reference true values. These points are pre-calibrated using a high-precision CMM. The angular values derived from the measured coordinates on the MBC are then compared with the reference true to establish an angular error equation.

According to the angle information composed of the initial measurement points in the MBC, the arccosine function is given by

(16) $$\theta_{i}=\arccos\left(\frac{a\cdot b}{\left\|a\right\|\left\|b\right\|}\right)$$

where $\theta_{i}$ is the angle between the vectors ***a*** and ***b***.

Next, the prior angle values are selected as the reference true values. The angular values derived from the field measurements of each target point are then subtracted from the reference true values to form the angular error equation. Taking angle $\theta_{ai}$ as an example, we apply a Taylor expansion to Equation (16), neglecting second-order and higher-order terms, resulting in the linearized equation for the angular constraint:

(17) θ^ai=θai0+∂θai∂x˜s1mDΔx˜s1mD+∂θai∂y˜s1mDΔy˜s1mD+∂θai∂zs1mDΔz˜s1mD+⋯+∂θai∂z˜snmDΔz˜snmD

where Δx˜simD,Δy˜simD,Δz˜simD represent the coordinate corrections of the initial measurement points, the nominal angle ${\overset{⌢}{\theta }}_{i}$ is calibrated by CMM, and ${\overset{⌢}{\theta }}_{i}^{0}$ is the measured angle.

The angular error is characterized by the following mathematical expression, which quantifies the relationship between measurement variables and angular deviation:

(18) $$v_{ai}={\hat{\theta }}_{ai}-\theta_{ai}^{0}$$

To further facilitate analysis, the above equation is reformulated into a matrix form as follows:

(19) $$W_{a}=B_{a}\Delta {\tilde{X}}_{a}-d_{a}$$

where ΔX˜a=Δx˜s1mD,Δy˜s1mD,⋯,Δz˜snmDT denotes the vector of coordinate corrections; $d_{a}$ represents the angular error vector; $B_{a}$ is the coefficient matrix.

According to the principle of least squares adjustment, the normal equations are derived as follows:

(20) $$\left({\left(B_{a}\right)}^{T}U_{a}B_{a}\right)\Delta {\tilde{X}}_{a}={\left(B_{a}\right)}^{T}U_{a}d_{a}$$

where $U_{a}$ is the weight matrix.

In the paper, the ridge estimation method [29] was employed to calculate the optimal parameters. As a result, after compensating for measurement errors, an optimized single virtual point P˜gimS is derived from the adjusted measurement points.

### 3.2. Accurate Calibration of Robot Kinematics and Hand-Eye Parameters

Discrepancies between the theoretical and actual kinematic parameters of the robot lead to deviations in the end-effector’s pose. Additionally, the robot motion involved in the initial hand-eye calibration process introduces inevitable positioning errors, further compromising calibration accuracy. Measurement errors from the 3D scanner also impose constraints on improving calibration precision. To address these issues, this section presents a joint calibration method based on a stereo target, aiming to simultaneously compensate for both hand-eye and kinematic parameter errors. Specifically, a distance error model is derived for associating the parameter errors with the deviations in the measured coordinates of the single virtual point. By reducing the impact of 3D scanner measurement noise and kinematic deviations, the proposed approach significantly enhances the calibration accuracy of the integrated robot-scanner system.

Assume that PcmS=xcmS,ycmS,zcmS,1T represents the homogeneous coordinates of a measurement point in the SCS, and the corresponding measurement result in the BCS can be denoted as

(21) PgimB=HEiBHSEPgimS

where $H_{Ei}^{B}$ and $H_{S}^{E}$ represent the theoretical values of the robot end-effector pose matrix and the hand-eye transformation matrix, respectively.

Considering the influences of hand-eye calibration errors, robotic kinematic parameter deviations, and 3D scanner measurement errors, the actual position of the measurement point in the BCS can be expressed as

(22) PcirB=HEiB+ΔHEiBHSE+ΔHSEPcmS+ΔPcmS

where ΔPcmS=ΔxcmS,ΔycmS,ΔzcmS,0T represents the 3D scanner measurement error, $\Delta H_{Ei}^{B}$ denotes the robotic end-effector pose error, and $\Delta H_{S}^{E}$ corresponds to the hand-eye calibration error.

By subtracting Equation (22) from Equation (21) and neglecting higher-order terms beyond the second order, the deviation between the actual and measured values of the point in the BCS is obtained:

(23) dPcB=HEiBHSEΔPcmS+HEiBΔHSEP⌢cmS+ΔHEiBHSEP⌢cmS

where P⌢cmS=PcmS+ΔPcmS is the corrected coordinates of the scanned measurement point. The first term on the right-hand side can be expressed as

(24) HEiBHSEΔPcmS=HEiBHSEΔxcmS,ΔycmS,ΔzcmS,0T=LiSΔTcmS

According to differential kinematics, the hand-eye relationship error can be expressed as

(25) $$\Delta H_{S}^{E}=H_{S}^{E}\delta H_{S}^{E}$$

where $\delta H_{S}^{E}$ is the differential operator.

Thus, the second term on the right-hand side of Equation (23) can be simplified as

(26) HEiBΔHSEP⌢cmS=HEiBHSEδHSEP⌢cmS=MiΔS

Similarly, the error model for the robot end-effector pose can be expressed as

(27) ΔHEiBHSEP⌢cmS=NiΔE=NiJiΔX

where P⌢cmB=HEiBHSEP⌢cmS represents the coordinates of the corrected scan measurement point transformed into the robot base coordinate system, and $\Delta E_{i}=J_{i}\Delta X$ represents the robot end-effector pose error model from Equation (4).

By neglecting second-order high-order terms, the hand-eye relationship model incorporating 3D scanner measurement errors and robot kinematic parameter errors is obtained as follows:

(28) dPcB=LiSΔTcmS+MiΔS+NiJiΔX=LiSΔTcmS+GiΔK

where $G_{i}=\left[\begin{array}{cc} M_{i} & N_{i}J_{i} \end{array}\right]$ is the coefficient matrix corresponding to the *i*-th calibration pose of the robot, while $\Delta K={\left[\begin{array}{cc} \Delta S & \Delta X \end{array}\right]}^{T}$ is a vector comprising system parameter errors, including hand-eye parameter errors and robot kinematic parameter errors.

**Figure 4:**
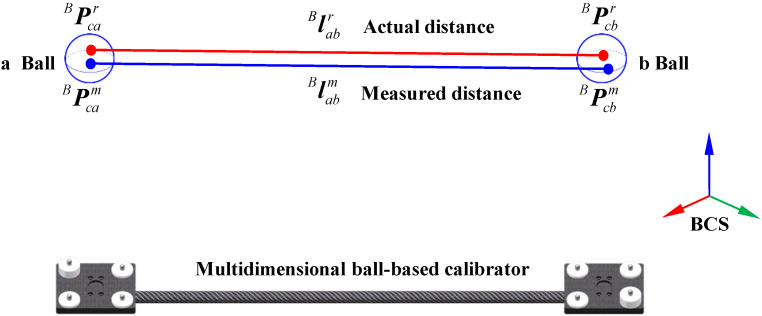
Schematic of distance error.

Building upon the preceding research, a system parameter identification model is further developed based on distance error analysis. In Euclidean space, the theoretical distance between two measured points should remain invariant across different coordinate systems. However, discrepancies arise between the theoretical and measured distances of two points in the BCS due to 3D scanner measurement errors, inaccuracies in hand-eye parameters, and deviations in robot kinematic parameters—collectively referred to as distance errors. In this paper, the distance error is defined as the difference between the calibrated distance and the measured distance between the center points of two spheres on the stereo target, as illustrated in Figure 4.

Let PcarB=xcarB,ycarB,zcarB and PcbrB=xcbrB,ycbrB,zcbrB represent the actual coordinates of points *a* and *b* on the stereo target in the BCS. Let PcamB=xcamB,ycamB,zcamB and PcbmB=xcbmB,ycbmB,zcbmB represent the measured coordinates of the corresponding points. Similarly, let labrB be the actual distance vector between the two points and labmB be the measured distance vector. The error vectors between the measured and actual values of the two points are denoted as dPcaB and dPcbB, respectively. Then, we have

(29) labrB=PabrB−PabrBlabmB=PabmB−PabmBdPcaB=PcarB−PcamBdPcbB=PcbrB−PcbmB

Thus, the distance error $\Delta l_{ab}$ between the two points can be expressed as

(30) Δlab=Δlab=labrB−labmB

where $\Delta l_{ab}$ is the distance error vector.

Then, we obtain

(31) Δlab=PcamB−PcbmBTlabmB×dPcbB−dPcaB

Substituting Equation (28) into the above expression yields:

(32) Δlab−PcamB−PcbmBTlabmBLbSΔTcbmS−LaSΔTcamS︸Δl=PcamB−PcbmBTlabmBGb−Ga︸QΔK

To refine the kinematic model and hand-eye transformation matrix for improved end-effector scanning accuracy, the position of the stereo target was varied, and the above process was repeated multiple times to establish a system of linear equations. The Levenberg-Marquardt (L-M) algorithm was then employed to solve for system parameter errors, resulting in a more accurate representation of the system.

## 4. Experimental and Discussion

**Figure 5:**
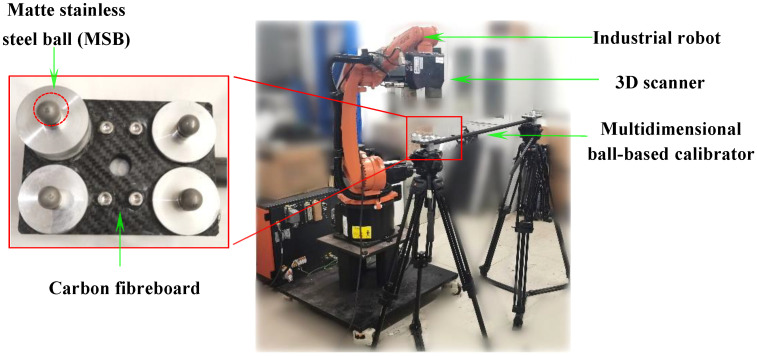
Proposed flexible 3D scanning system.

To verify the effectiveness of the proposed calibration method, a robot-scanner experimental system was established, and corresponding validation experiments were conducted. As illustrated in Figure 5, the system mainly comprises a KUKA-KR industrial robot (KUKA Robotics, Augsburg, Germany) with a repeatability of 0.04 mm and a structured light 3D scanner. The scanner, a binocular fringe projection system from the LMI Gocator 3210 (LMI Technologies Inc., Burnaby, BC, Canada), has its key performance specifications listed in Table 1. The calibration experiments are presented were carried out in Section 4.1, while Section 4.2 assesses the calibration accuracy by measuring a metric artifact in accordance with VDI/VDE 2634 Part 3 [30]. All experiments were conducted in a stable laboratory environment, with the indoor temperature varying between 22 °C and 23 °C and the relative humidity ranging from 55% to 60%.

For precise calibration and performance evaluation, a customized multidimensional ball-based calibrator (MBC) with a length of 1000 mm was designed specifically for 3D scanner measurements. As shown in Figure 5, the calibrator is made of carbon fiber reinforced polymer (CFRP) and integrates eight matte stainless steel balls (MSBs). Each MSB has a diameter of 12.70 mm and a roundness deviation of less than 0.002 mm. The spatial relationships among all balls were pre-calibrated using a Zeiss PRISMO coordinate measuring machine (Carl Zeiss Industrielle Messtechnik GmbH, Oberkochen, Germany), a high-precision CMM widely used in industrial metrology. In this study, the maximum inter-sphere distance is 1006.863 mm. According to the manufacturer, the machine provides a length measurement error (MPE) of

$$E_{0}=\left(0.9+L/350\right)\;\mu m$$

where *L* is the measured length in millimeters. For our typical inter-sphere distances, this corresponds to a measurement error below 1.5 μm. A Type A uncertainty analysis based on repeated measurements, combined with Type B estimates (instrument specs and environmental control), leads to a conservative combined uncertainty of ±2 μm (k = 2) for each sphere center distance. These uncertainty estimates provide a reliable reference for evaluating the calibration accuracy of the proposed method.

### 4.1. Calibration Experiments

The accuracy of hand-eye calibration is known to be highly sensitive to the spatial distribution and diversity of the robot poses used during the calibration process. To ensure robust and accurate hand-eye calibration, we adopt the following pose selection strategy: First, we generate at least 12–15 poses with varied orientations and positions covering a large portion of the robot’s reachable workspace. Then, the selected poses avoid alignment along a single axis (collinearity) or within a single plane, thereby increasing the observability of rotational and translational parameters. Furthermore, each pose ensures that the 3D scanner maintains a consistent view of the calibration target, avoiding occlusion and maintaining sufficient overlap for accurate point cloud registration. Specifically, the range of end-effector orientations is selected to include rotations about different axes, and translational displacements cover different spatial quadrants.

**Figure 6:**
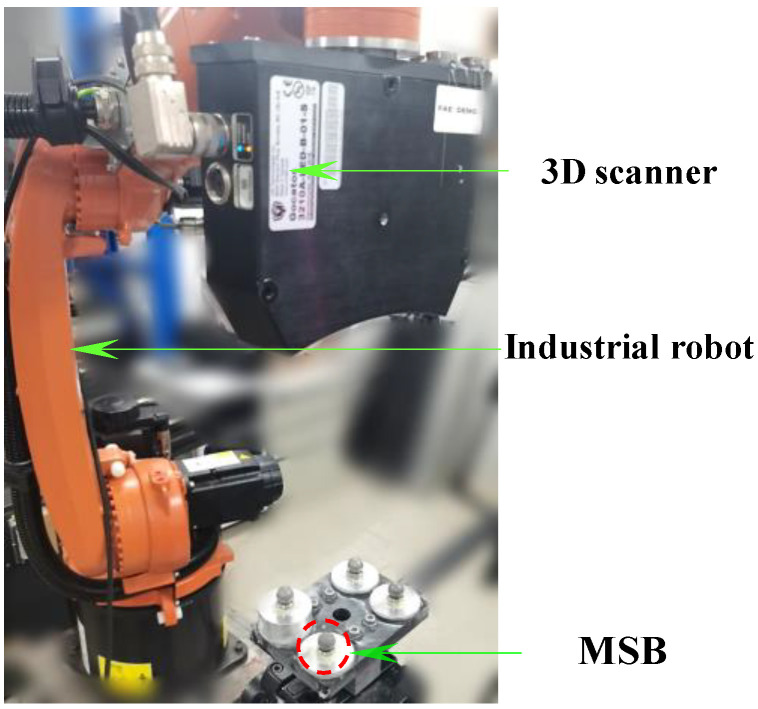
Experiment on the impact of robot posture on calibration accuracy.

To validate the effectiveness of this strategy, we conducted a contrast experiment in which three different pose configurations were tested, as shown in Figure 6. Specifically, in this study, we compare the radius error of 0.5-inch MSBs obtained using (i) collinear poses, (ii) planar poses, and (iii) spatially distributed poses, under the same conditions. The experimental steps are as follows: First, the hand-eye transformation matrix was estimated under three different robot poses using a calibration method based on a single sphere [18]. The resulting point cloud data of the measured MSB were then transformed into the robot coordinate frame. The radius of the MSB was reconstructed and compared with its pre-calibrated reference value to compute the radius error of the MSB for each pose, as summarized in Table 2. These results confirm that greater pose diversity significantly improves calibration accuracy and reduces numerical instability.

Based on the comprehensive analysis of robot pose diversity and its influence on calibration accuracy, we proceeded to apply the proposed calibration method using the optimized pose. The following section details the setup, procedure, and results of the calibration experiments conducted to validate the effectiveness and robustness of our method: First, an initial hand-eye calibration experiment was conducted following the method introduced in Section 3.1. A multidimensional ball-based calibrator was positioned within the robot’s workspace, and the robot was programmed to execute six translational movements and six orientation changes. To prevent singularities, the end-effector was translated along the X, Y, and Z axes of the robot’s base coordinate frame during the six translational movements. At each pose, the 3D scanner performed multiple measurements of the target spheres on the stereo target. Using the first six sets of measurements, the rotation matrix $R_{S}^{E}$ was computed according to Equation (7). Subsequently, the translation vector $T_{S}^{E}$ was determined from the remaining six sets of data, yielding the initial estimate of the hand-eye transformation matrix.

Subsequently, a high-precision hand-eye calibration experiment was performed. The MBC, mounted on a tripod, was successively placed at eight distinct vertical positions within the workspace. At each position, the robot’s end-effector executed eight unique orientation changes. A 3D scanner was employed to capture the target points and extract the coordinates of the sphere centers. Based on these measurements, a system parameter error identification model was constructed to estimate the kinematic parameter errors, as presented in Table 3. The refined hand-eye transformation matrix was then accurately determined through this high-precision calibration process, as shown below:

$${\overset{⌢}{H}}_{S}^{E}=\left[\begin{array}{cccc} -0.0171 & -0.9998 & -0.0053 & 1.2874\\ -0.9999 & 0.0173 & -0.0022 & -0.5842\\ 0.0003 & 0.0056 & -1.0001 & 264.7846\\ 0 & 0 & 0 & 1 \end{array}\right]$$

The entire calibration procedure is highly automated and completes within 8 min, enabling efficient on-site recalibration of the system, when necessary, without interrupting standard workflow.

### 4.2. Accuracy Evaluation of Accurate Calibration

**Figure 7:**
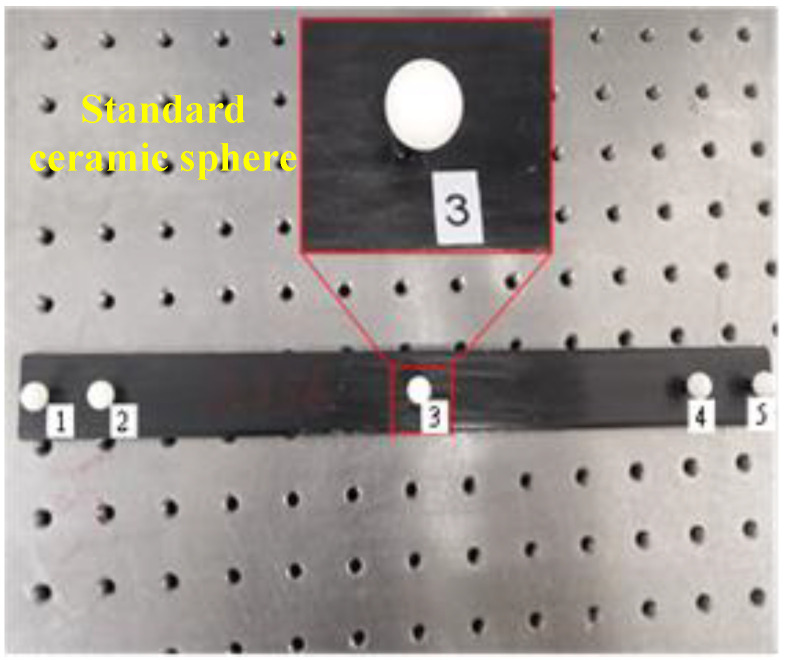
Standard scale used for calibration accuracy verification.

To evaluate the calibration accuracy, a standard scale was utilized, as shown in Figure 7. The scale primarily consists of several homogeneous and precision-grade ceramic spheres with an approximate diameter. Each standard ceramic sphere has a diameter of 10.010 mm and a roundness deviation of 0.001 mm. The center-to-center distance between two selected spheres was pre-calibrated using a coordinate measuring machine (CMM) [30], yielding a result of D15 = 299.546 mm.

Therefore, the errors between the measured and actual distances of sphere centers on the standard scale were computed at ten different spatial positions, using system parameters obtained both before and after calibration. The corresponding results are presented in Table 4. Following calibration, the maximum (MPE) and mean errors (ME) were reduced from 1.053/0.814 mm to 0.421/0.373 mm, respectively. These results satisfy the accuracy requirements for scanning critical and hard-to-reach features, thereby confirming the reliability and effectiveness of the proposed method.

To better understand the accuracy and robustness of the proposed calibration method, we performed a qualitative analysis of the major sources of error involved in the hand-eye and kinematic parameter calibration process. These error sources can be categorized into the following three aspects: (i) robot pose repeatability and mechanical uncertainty. Although the KUKA industrial robot employed in this study offers a high repeatability of ±0.04 mm, it is not immune to small deviations caused by joint backlash, compliance, or control jitter. These minor inconsistencies directly affect the transformation between the robot base and end-effector and propagate into the final calibration results; (ii) 3D scanner measurement error. The LMI Gocator 3 series structured-light scanner used in this study has a nominal accuracy better than 0.035 mm. However, in practical use, factors, such as surface reflectivity, ambient lighting, and sensor-to-target distance, may introduce fluctuations in the measured point cloud, resulting in uncertainty in the extracted geometric features; and (iii) pose selection and geometric observability.

In summary, while sensor noise and sphere fitting errors primarily affect the input data quality, the pose distribution and optimization formulation govern the observability and convergence of the calibration. These factors jointly determine the final calibration accuracy.

**Figure 8:**
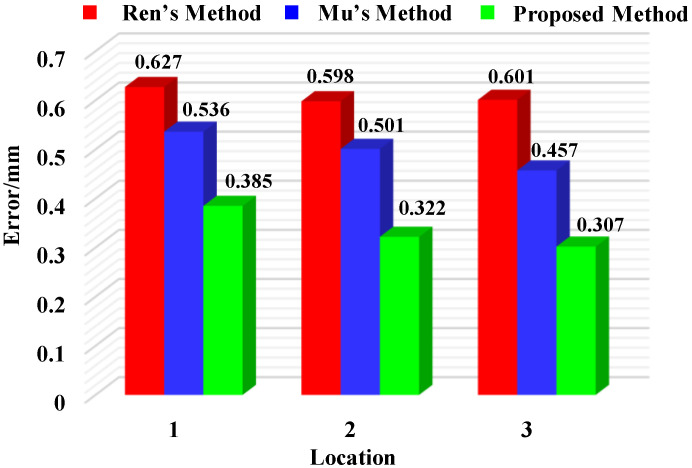
Mean sphere spacing errors at different locations.

To further validate the effectiveness of the proposed calibration method, mean sphere spacing errors at multiple positions were evaluated using the methods proposed by Ren [18], Mu [25], and this study, respectively, as shown in Figure 8. When using Ren’s method, the mean sphere spacing errors at positions 1 to 3 were 0.627 mm, 0.598 mm, and 0.601 mm, respectively. In comparison, Mu’s method yielded average errors of 0.536 mm, 0.501 mm, and 0.457 mm for the same positions. By contrast, the proposed method achieved lower mean errors of 0.385 mm, 0.322 mm, and 0.307 mm at positions 1 to 3. These experimental results demonstrate the superior accuracy of the calibration technique developed in this study.

## 5. Conclusions

In this study, a flexible measurement system integrating a fringe-projection 3D scanner with an industrial robot was developed to enable efficient measurement of large-scale surfaces. To improve overall measurement accuracy, we proposed an enhanced calibration method employing a multidimensional ball-based calibrator to simultaneously compensate for hand-eye transformation and robot kinematic errors. Calibration and validation experiments were carried out, and the results indicate that the maximum and average measurement errors were reduced from 1.053 mm and 0.814 mm to 0.421 mm and 0.373 mm, respectively, thereby confirming the effectiveness of the proposed method. The entire calibration workflow is highly automated and can be executed in less than 8 min. This facilitates practical on-site recalibration during system deployment.

Future research will further investigate the pose selection strategy, including experimental evaluations to assess how pose diversity influences calibration accuracy. In addition, a detailed analysis of potential error sources will be conducted. Efforts will also be made to integrate the current system with an automated guided vehicle (AGV) platform, enabling initial measurement trials on large-scale industrial parts. Particular emphasis will be placed on quantitatively validating the measurement accuracy for these large components under real-world conditions.

## Figures and Tables

**Table 1:** Main parameters of LMI 3D scanner.

Parameter	Value	Parameter	Value
Field of view (mm)	71 × 98~100 × 154	Measuring distance (mm)	165
Scanning speed (HZ)	4	Resolution in XY direction (µm)	60~90
VDE accuracy (mm)	0.035	Optical source	Blue LED light

**Table 2:** Measurement result of MSB (unit: mm).

Pose Configuration	Average Radius	Average Radius Error
Collinear poses	7.112	0.756
Planar poses	6.993	0.637
Spatially distributed poses	6.397	0.412

**Table 3:** Identification results of robot parameter errors.

Link No.	$\Delta \theta_{i}$/°	$\Delta d_{i}$/mm	$\Delta a_{i}$/mm	$\Delta \alpha_{i}$/°	$\Delta \beta_{i}$
1	0.0206	0.4539	0.1428	0.0074	—
2	−0.0169	—	−0.2632	0.0022	0.0079
3	0.0309	−0.2168	0.0508	−0.0127	—
4	−0.0407	0.0687	0.0268	0.0014	—
5	0.0274	−0.0832	−0.0034	−0.0137	—
6	−0.0237	0.1041	0.0378	0.0293	—

**Table 4:** Sphere spacing errors before and after calibration (unit: mm).

No.	1	2	3	4	5	MPE	ME
Before	0.762	0.901	0.826	0.775	0.774	1.053	0.814
	6	7	8	9	10		
	1.053	0.775	0.813	0.728	0.735		
No.	1	2	3	4	5	MPE	ME
After	0.337	0.407	0.417	0.384	0.342	0.421	0.373
	6	7	8	9	10		
	0.421	0.363	0.374	0.351	0.341		

## Data Availability

The data presented in this study are available upon request from the corresponding authors.
